# Supplementary material for: Shigella type-III secretion system effectors counteract the induction of host inflammation and cell death
Source: EMBO J. 2025 Sep 10;44(21):6196–225. doi: 10.1038/s44318-025-00561-7 (PMC12583537; doi:10.1038/s44318-025-00561-7)
Supplement: Supplementary file 1 — Appendix [file 44318_2025_561_MOESM1_ESM.pdf]

Appendix for:

"*Shigella* type-III secretion system effectors counteract the induction of host inflammation and cell death"

Hiroshi Ashida[1,2,] *, Tokuju Okano[1], Tamako Iida[1], Poramed Onsoi[1], Chihiro Sasakawa[2], and Toshihiko Suzuki[1,] *

Appendix Figure S1……………………………………………………Page 2

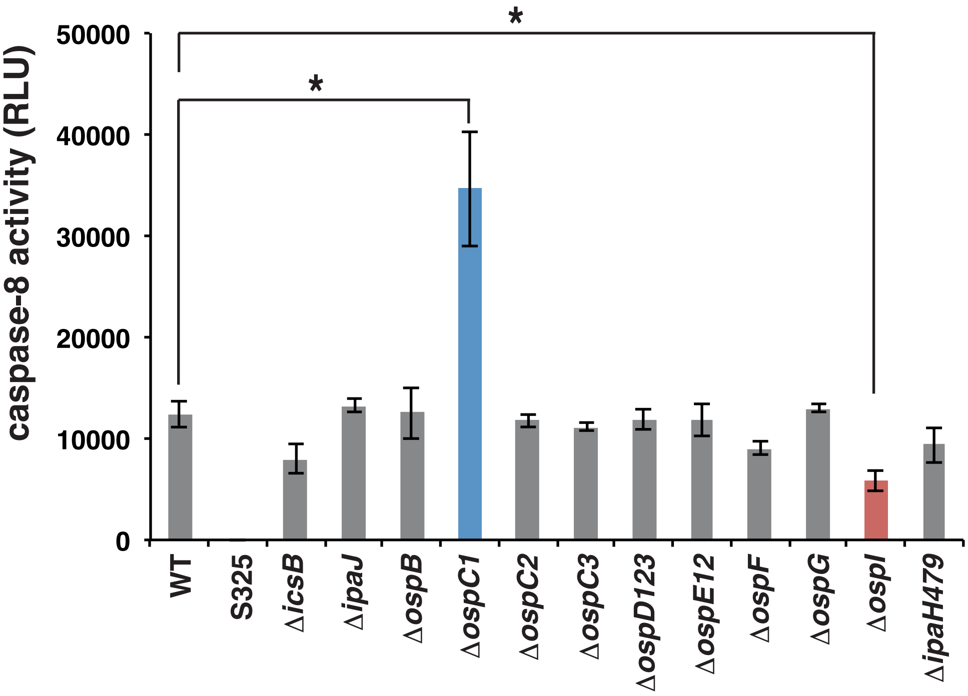

Appendix Figure S1. ***Shigella*** **OspI effector induces caspase-8 activation**

HT-29 cells were infected with the indicated *Shigella* strains and incubated for 8 h. Cells were harvested and subjected to measurement of caspase-8 activation. Caspase-8 activity is reported as relative light units (RLU) of infected samples, minus the value in uninfected samples.
Data are expressed as the mean ± SD from triplicate and representative of three independent experiments ($P = 0.0005$ for Δ*ospC1* and $P = 0.00218$ for Δ*ospI*; one-way ANOVA).
Data are considered significant when $P < 0.05$, with $^*P < 0.05$

Appendix Fig. S1
