# Supplementary figures and images for: Shigella type-III secretion system effectors counteract the induction of host inflammation and cell death
Source: EMBO J. 2025 Sep 10;44(21):6196–225. doi: 10.1038/s44318-025-00561-7 (PMC12583537; doi:10.1038/s44318-025-00561-7)

Fig. 1A

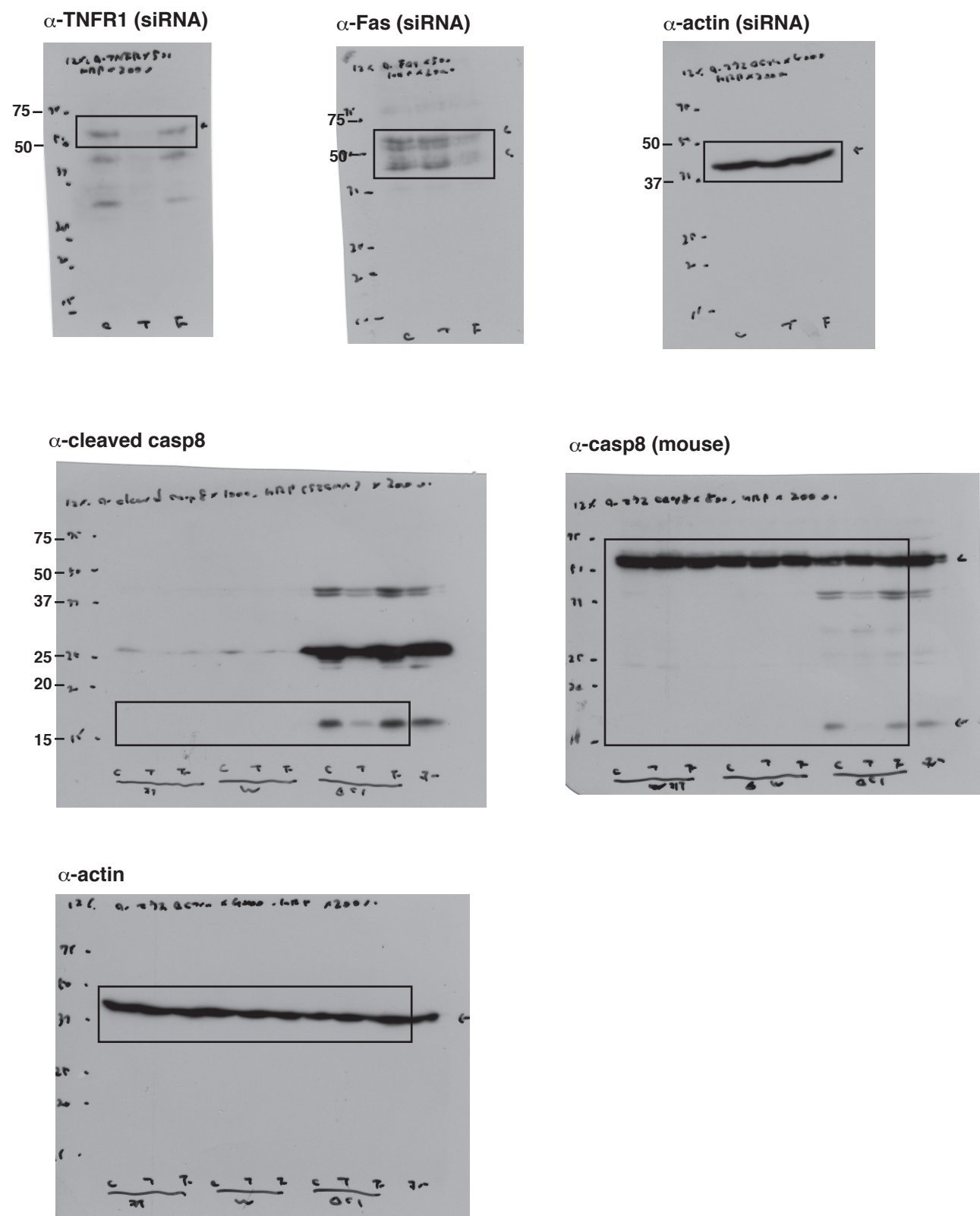

Source data for Fig. 1A

Supplement: Supplementary file 3 — Source data Fig. 1 [file 44318_2025_561_MOESM3_ESM.zip › Fig. 1/Source data for Fig. 1A/Source data for Fig. 1A.pdf]

Fig. 1B

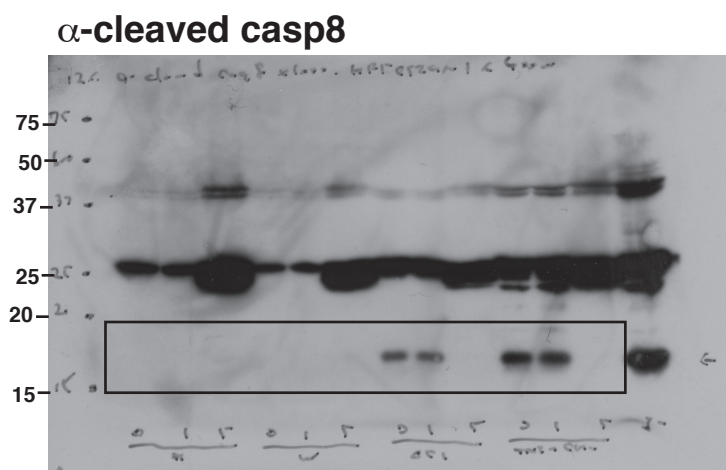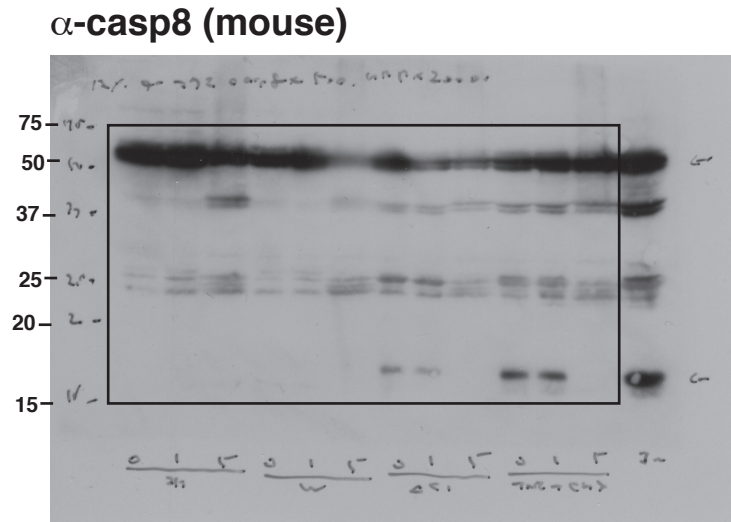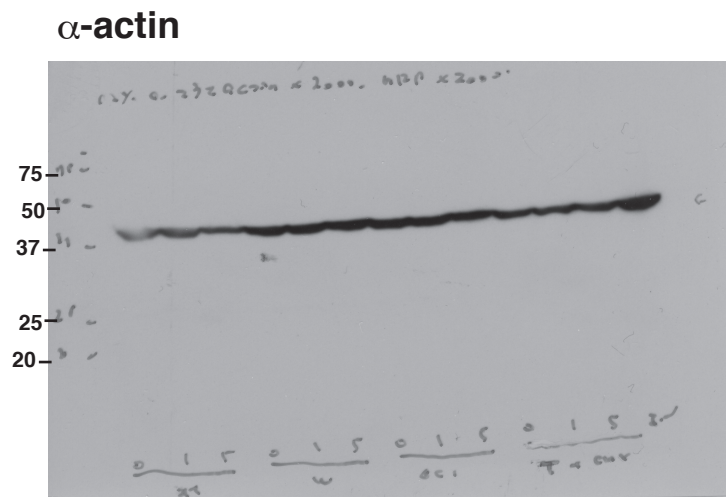

Source data for Fig. 1B

Supplement: Supplementary file 3 — Source data Fig. 1 [file 44318_2025_561_MOESM3_ESM.zip › Fig. 1/Source data for Fig. 1B/Source data for Fig. 1B.pdf]

Fig. 1C

$\alpha$ -cleaved casp8

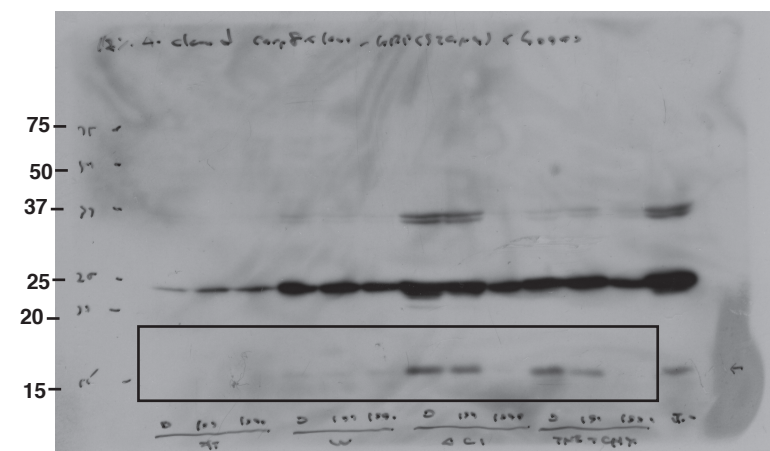

$\alpha$ -casp8 (mouse)

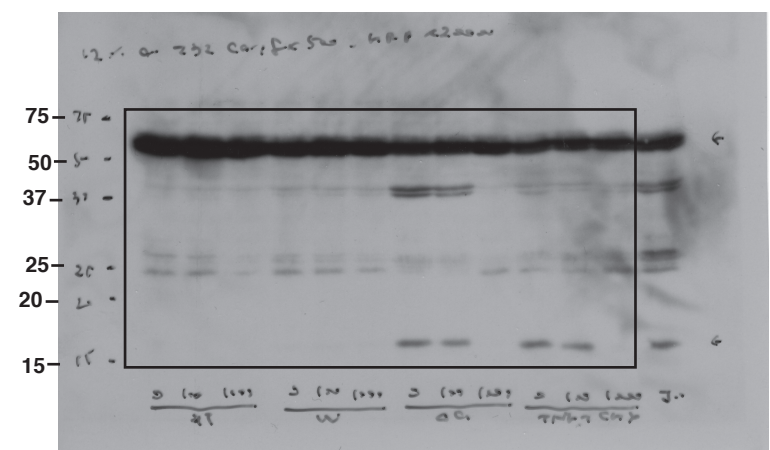

$\alpha$ -actin

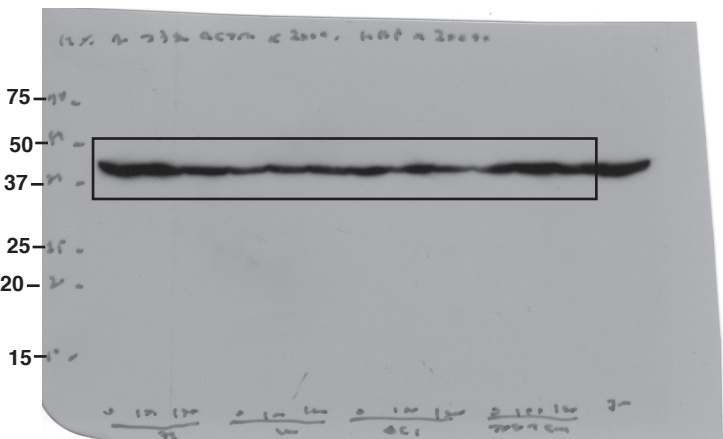

Supplement: Supplementary file 3 — Source data Fig. 1 [file 44318_2025_561_MOESM3_ESM.zip › Fig. 1/Source data for Fig. 1C/Source data for Fig. 1C.pdf]

**Fig. 1 E**

**$\alpha$ -cleaved casp8**

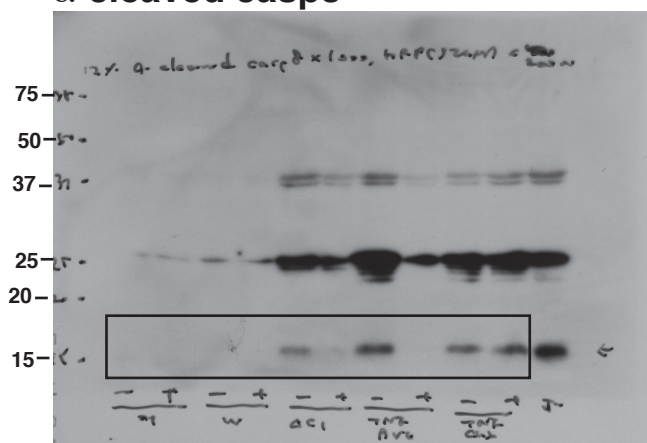

**$\alpha$ -casp8 (mouse)**

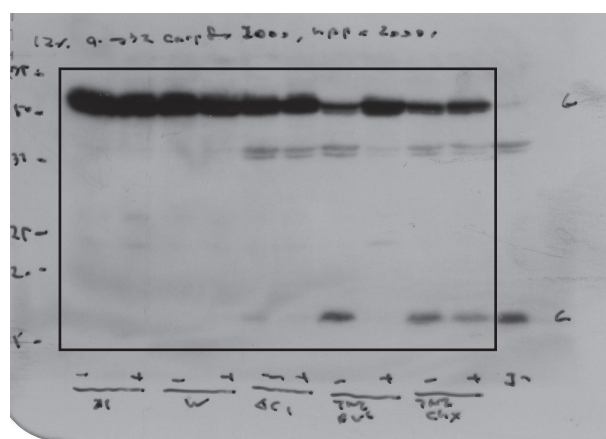

**$\alpha$ -actin**

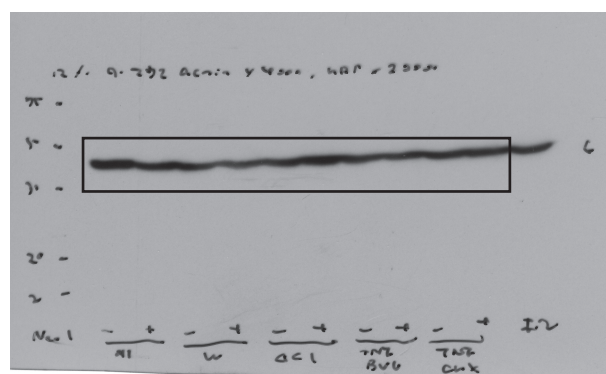

### Source data for Fig. 1E

Supplement: Supplementary file 3 — Source data Fig. 1 [file 44318_2025_561_MOESM3_ESM.zip › Fig. 1/Source data for Fig. 1E/Source data for Fig. 1E.pdf]

Fig. 1G

$\alpha$ -cleaved casp8

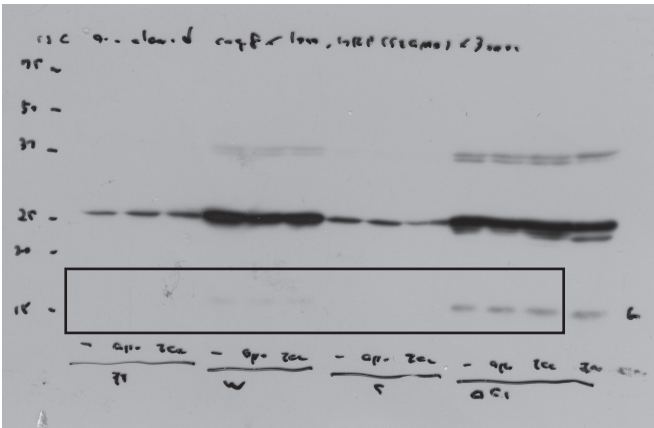

$\alpha$ -casp8 (mouse)

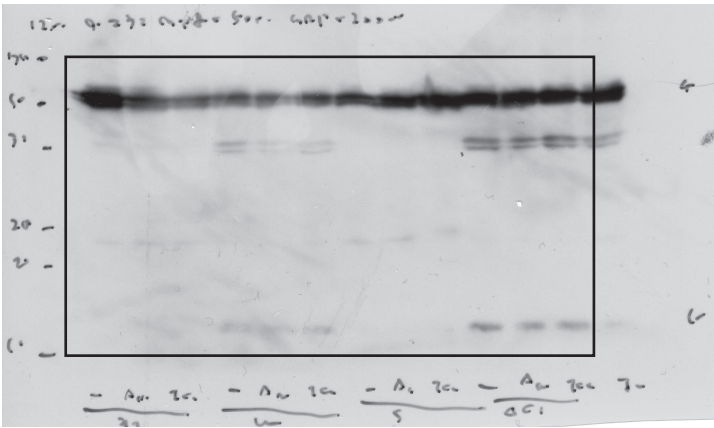

$\alpha$ -actin

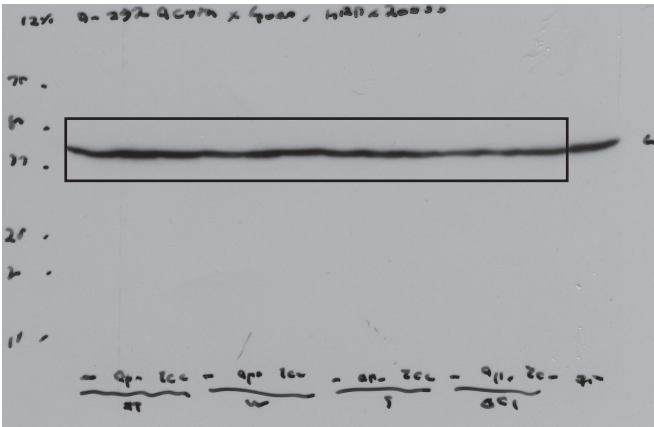

Supplement: Supplementary file 3 — Source data Fig. 1 [file 44318_2025_561_MOESM3_ESM.zip › Fig. 1/Source data for Fig. 1G/Source data for Fig. 1G.pdf]

Fig. 1H

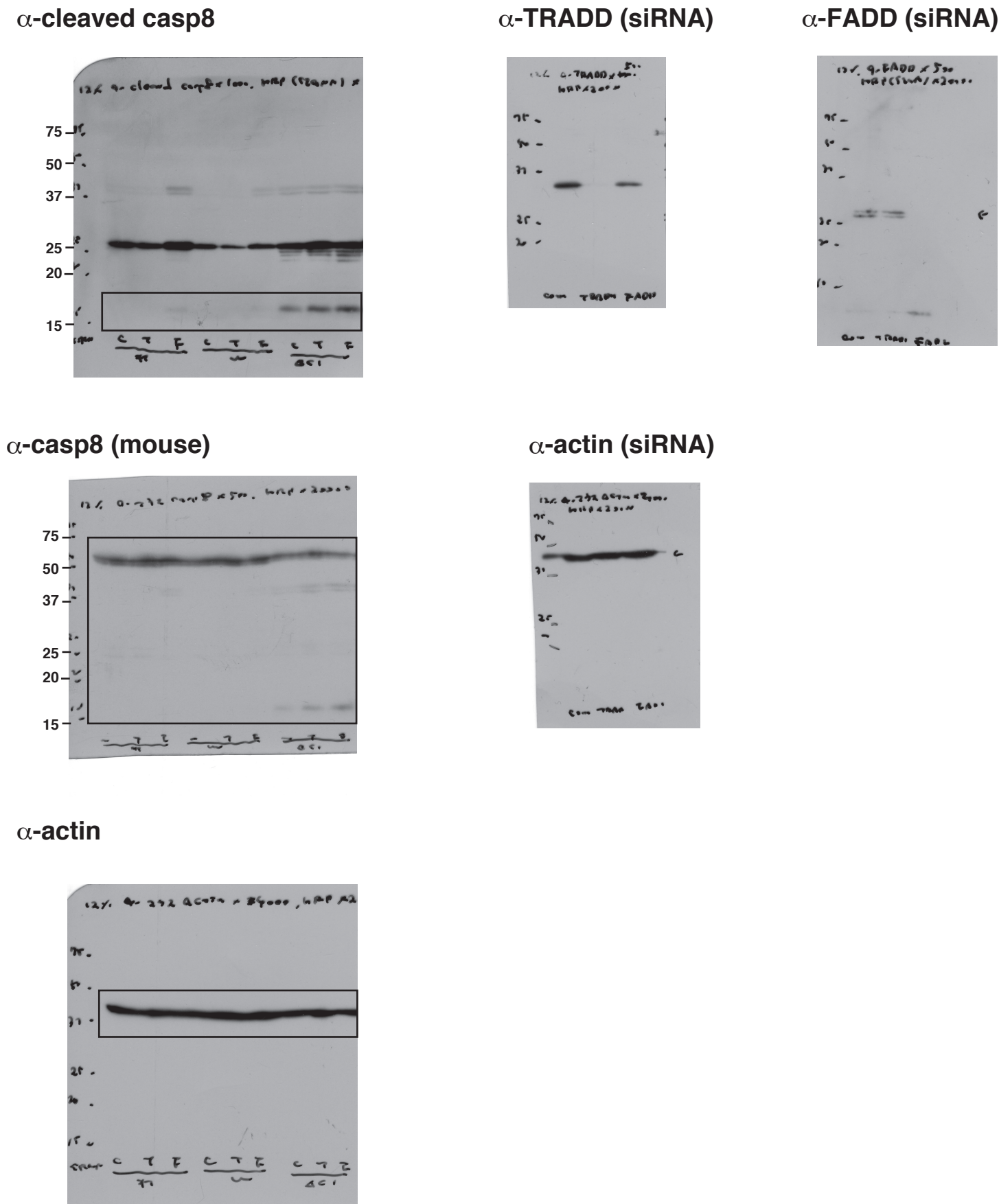

Supplement: Supplementary file 3 — Source data Fig. 1 [file 44318_2025_561_MOESM3_ESM.zip › Fig. 1/Source data for Fig. 1H/Source data for Fig. 1H.pdf]

Fig. 2G

$\alpha$ -cleaved casp8

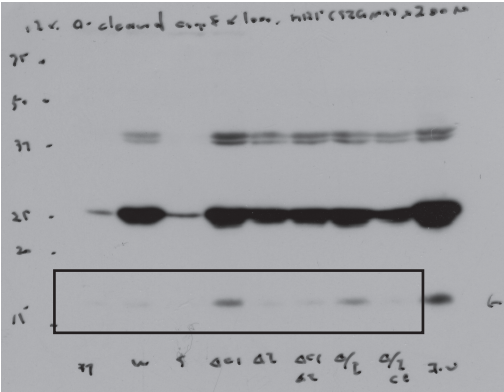

$\alpha$ -casp8 (mouse)

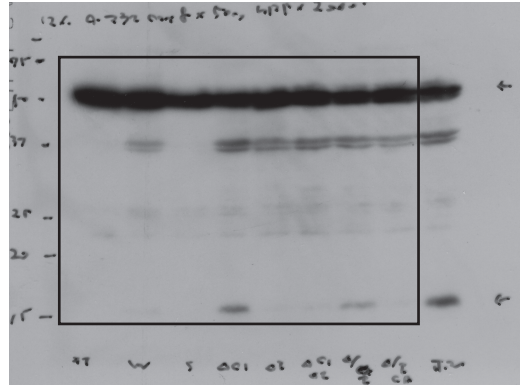

$\alpha$ -casp3

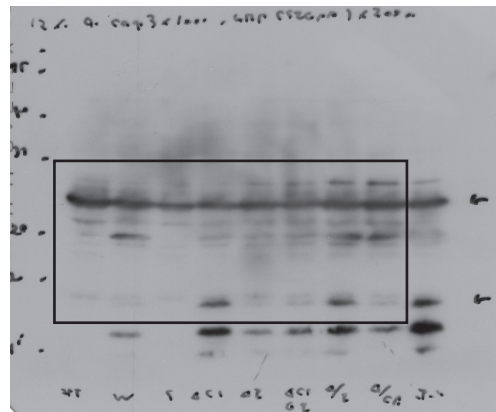

$\alpha$ -PARP

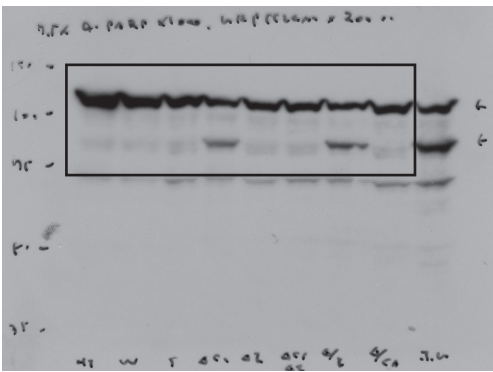

$\alpha$ -actin

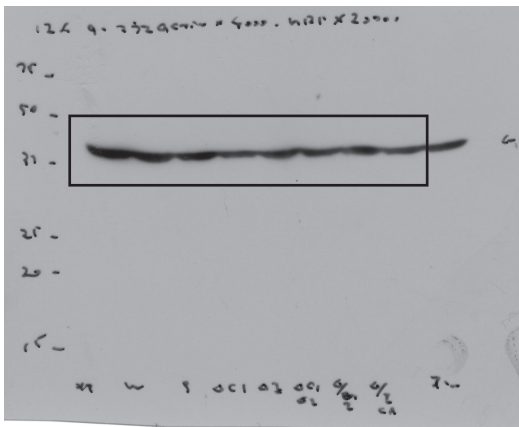

Source data for Fig. 2G

Supplement: Supplementary file 4 — Source data Fig. 2 [file 44318_2025_561_MOESM4_ESM.zip › Fig. 2/Source data for Fig. 2G/Source data for Fig. 2G.pdf]

**Fig. 3A**

**$\alpha$ -cleaved casp8**

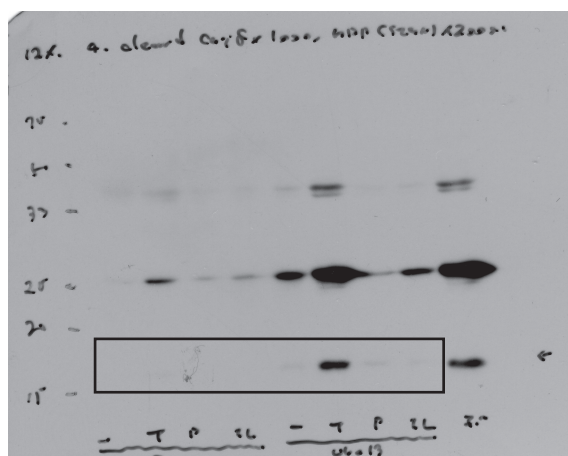

**$\alpha$ -casp8 (mouse)**

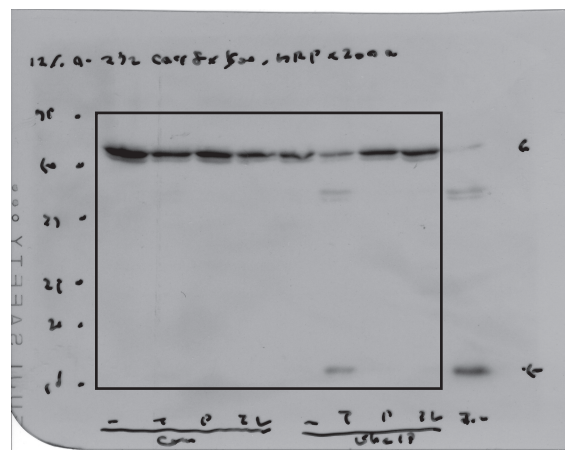

**$\alpha$ -casp3**

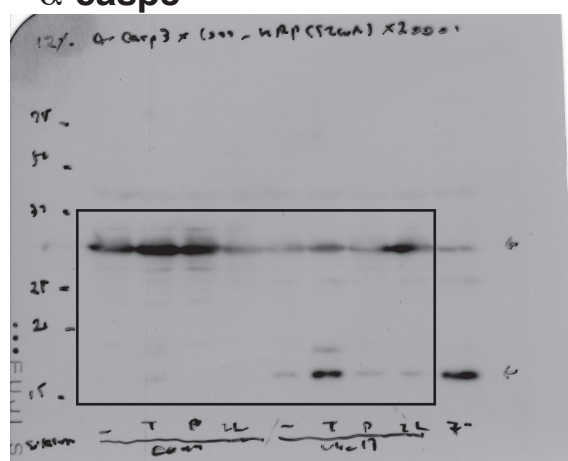

**$\alpha$ -PARP**

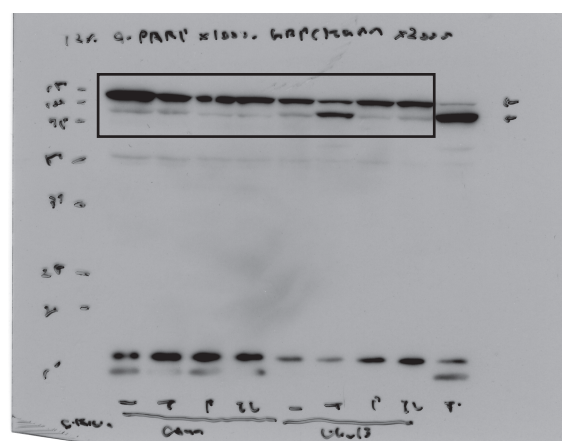

**$\alpha$ -Ubc13**

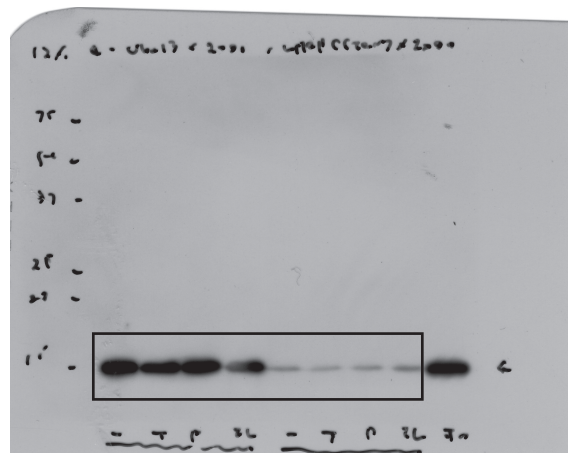

**$\alpha$ -actin**

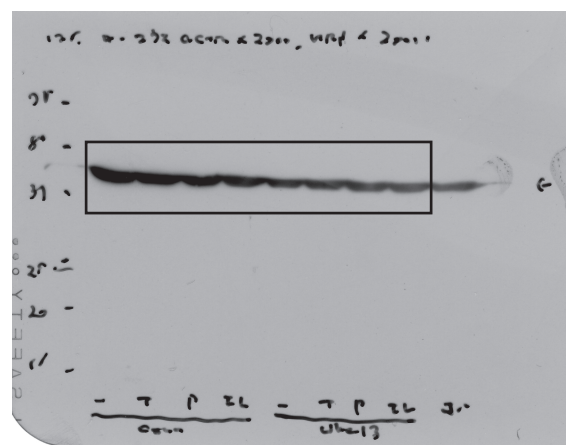

Supplement: Supplementary file 5 — Source data Fig. 3 [file 44318_2025_561_MOESM5_ESM.zip › Fig. 3/Source data for Fig. 3A/Source data for Fig. 3A.pdf]

Fig. 3C

$\alpha$ -cleaved casp8

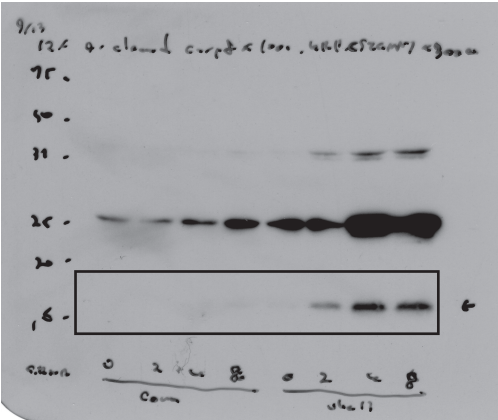

$\alpha$ -casp8 (mouse)

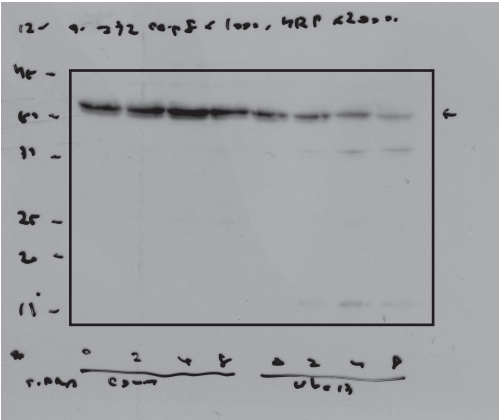

$\alpha$ -casp3

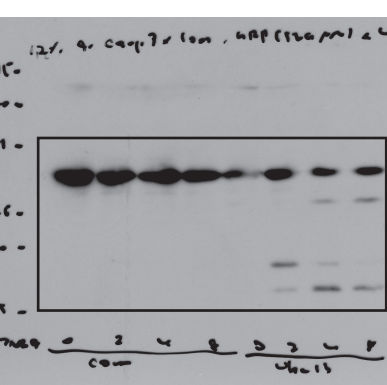

$\alpha$ -PARP

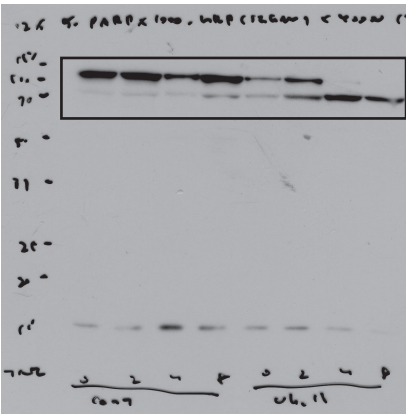

$\alpha$ -Ubc13

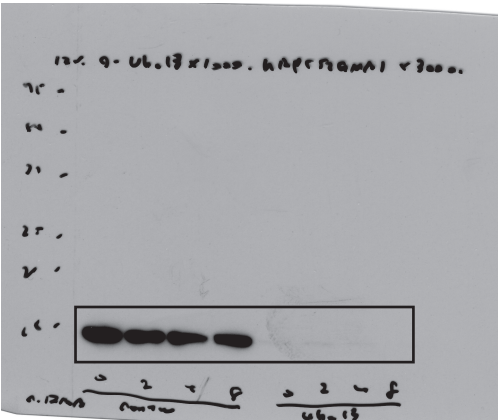

$\alpha$ -actin

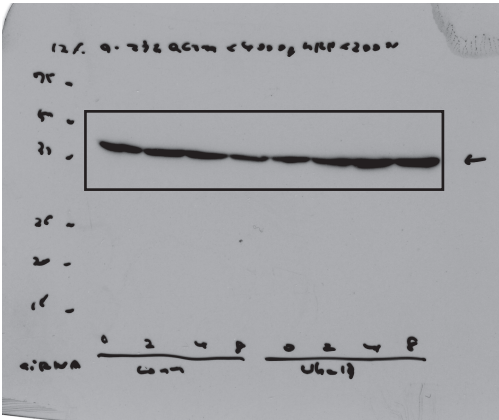

Supplement: Supplementary file 5 — Source data Fig. 3 [file 44318_2025_561_MOESM5_ESM.zip › Fig. 3/Source data for Fig. 3C/Source data for Fig. 3C.pdf]

**$\alpha$ -cleaved casp8**

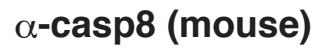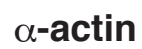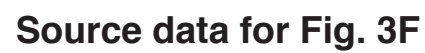

Supplement: Supplementary file 5 — Source data Fig. 3 [file 44318_2025_561_MOESM5_ESM.zip › Fig. 3/Source data for Fig. 3F/Source data for Fig. 3F.pdf]

Fig. 3H

$\alpha$ -cleaved casp8

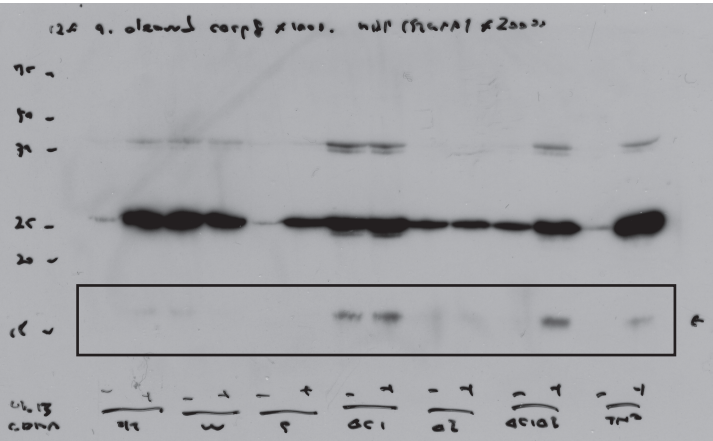

$\alpha$ -casp8 (mouse)

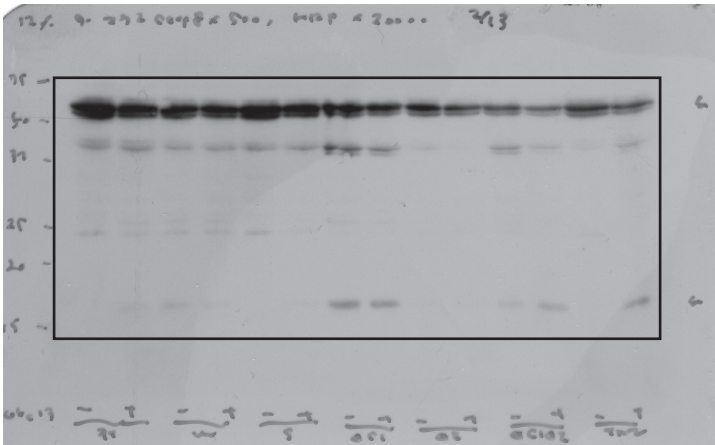

$\alpha$ -Ubc13

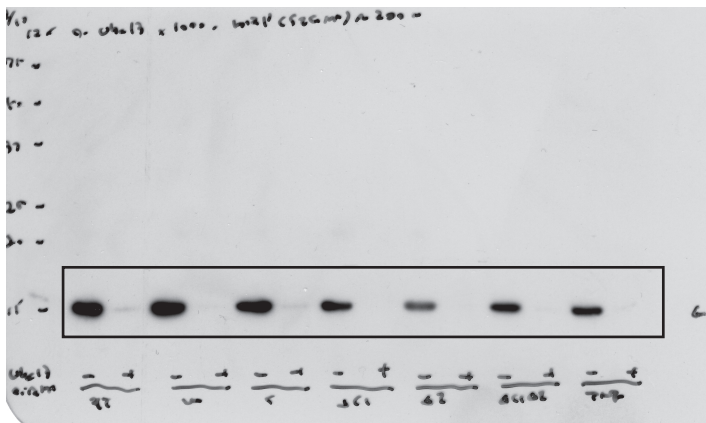

$\alpha$ -actin

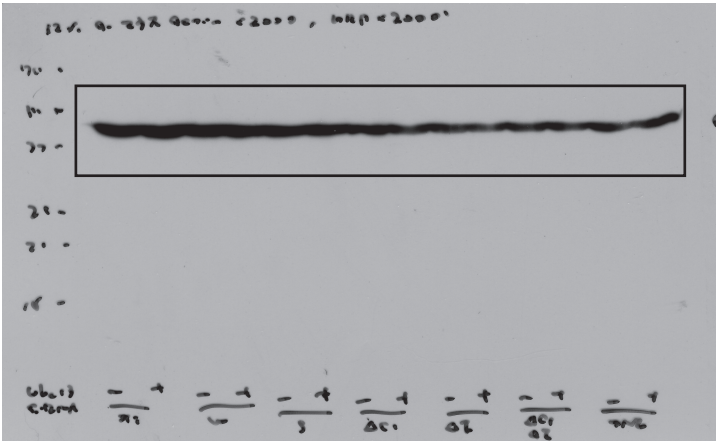

Source data for Fig. 3H

Supplement: Supplementary file 5 — Source data Fig. 3 [file 44318_2025_561_MOESM5_ESM.zip › Fig. 3/Source data for Fig. 3H/Source data for Fig. 3H.pdf]

Fig. 4B

$\alpha$ -cleaved casp8

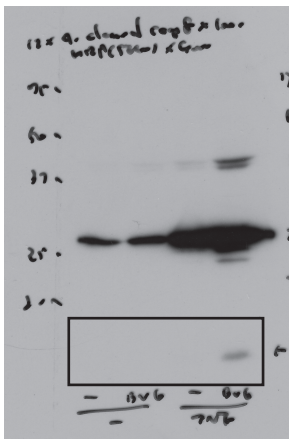

$\alpha$ -casp8 (mouse)

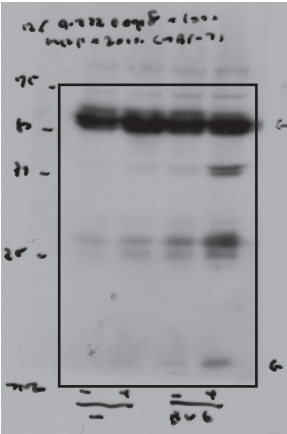

$\alpha$ -clAP1

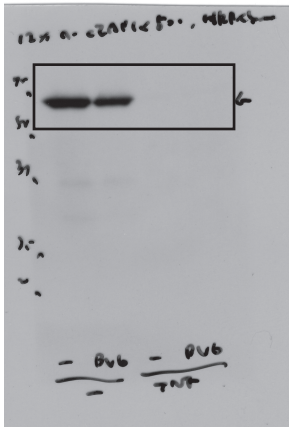

$\alpha$ -actin

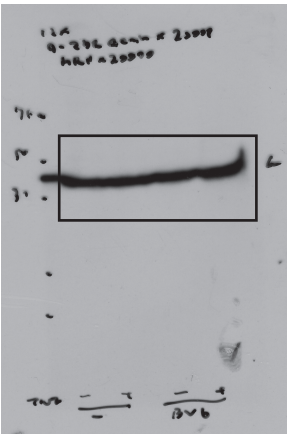

Source data for Fig. 4B

Supplement: Supplementary file 6 — Source data Fig. 4 [file 44318_2025_561_MOESM6_ESM.zip › Fig. 4/Source data for Fig. 4B/Source data for Fig. 4B.pdf]

Fig. 4C

$\alpha$ -cleaved casp8

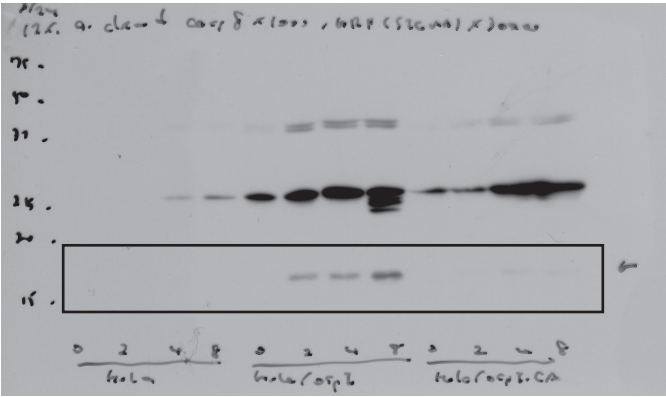

$\alpha$ -casp8 (mouse)

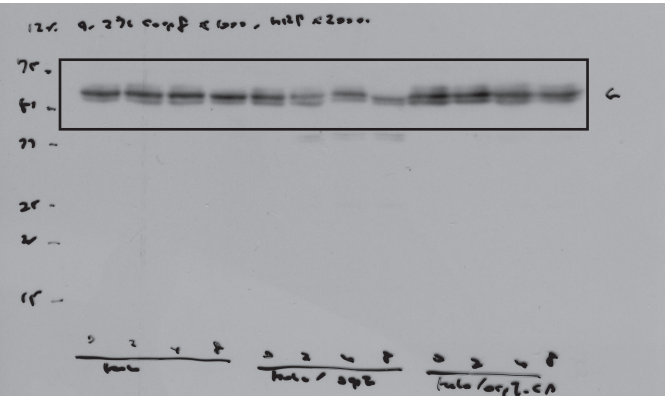

$\alpha$ -clAP1

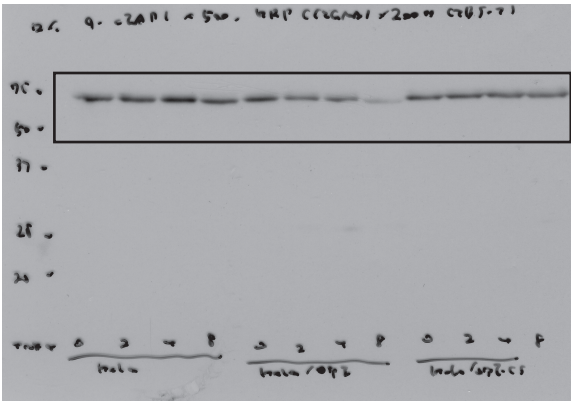

$\alpha$ -actin

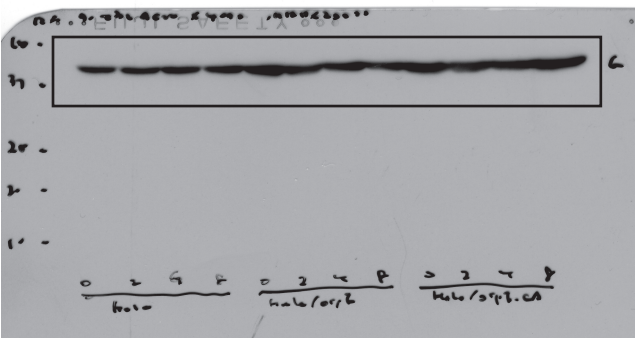

Source data for Fig. 4C

Supplement: Supplementary file 6 — Source data Fig. 4 [file 44318_2025_561_MOESM6_ESM.zip › Fig. 4/Source data for Fig. 4C/Source data for Fig. 4C.pdf]

Fig. 4D

$\alpha$ -cleaved casp8

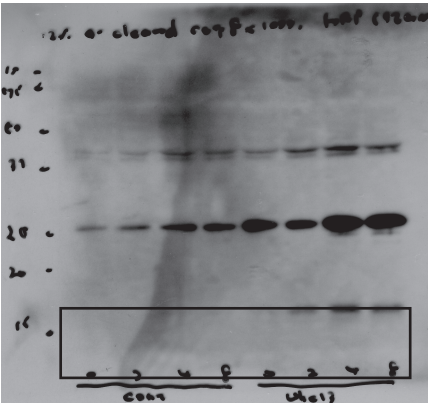

$\alpha$ -clAP1

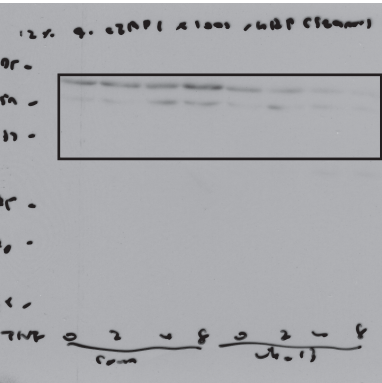

$\alpha$ -Ubc13

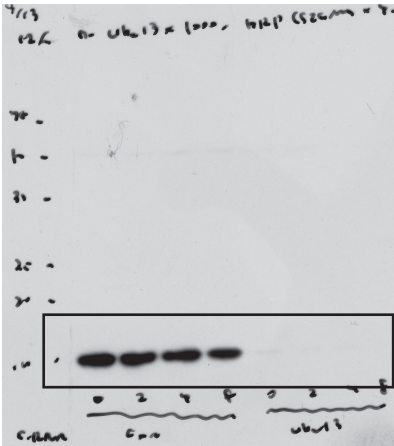

$\alpha$ -actin

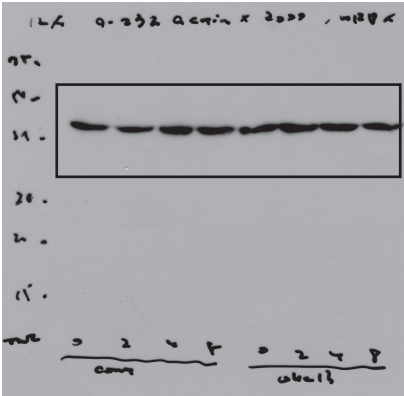

Source data for Fig. 4D

Supplement: Supplementary file 6 — Source data Fig. 4 [file 44318_2025_561_MOESM6_ESM.zip › Fig. 4/Source data for Fig. 4D/Source data for Fig. 4D.pdf]

Fig. 5A

$\alpha$ -ADP ribose

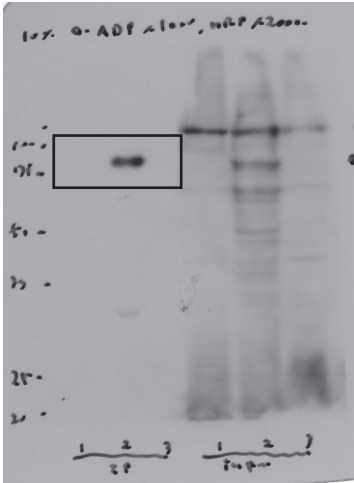

$\alpha$ -M2-FLAG

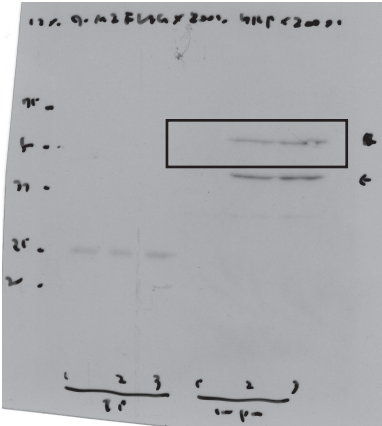

$\alpha$ -GFP

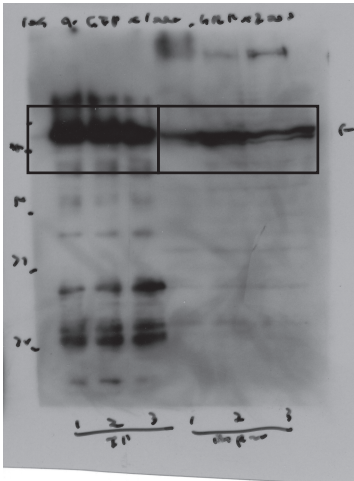

Source data for Fig. 5A

Supplement: Supplementary file 7 — Source data Fig. 5 [file 44318_2025_561_MOESM7_ESM.zip › Fig. 5/Source data for Fig. 5A/Source data for Fig. 5A.pdf]

Fig. 5B

$\alpha$ -ADP ribose

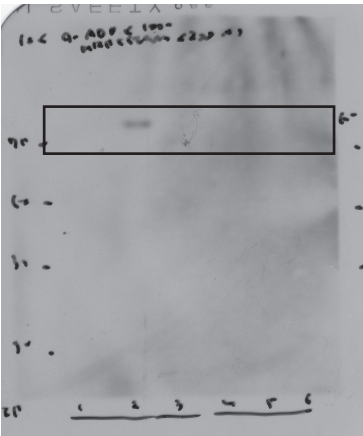

$\alpha$ -M2-FLAG

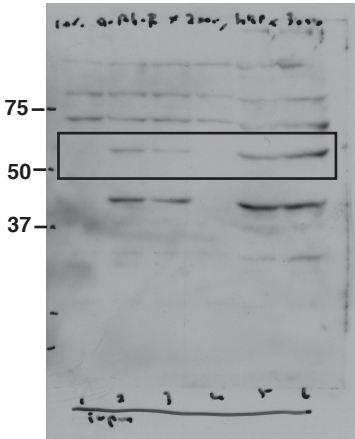

$\alpha$ -GFP (IP)

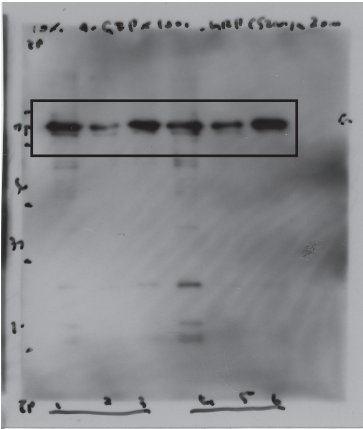

$\alpha$ -GFP (Input)

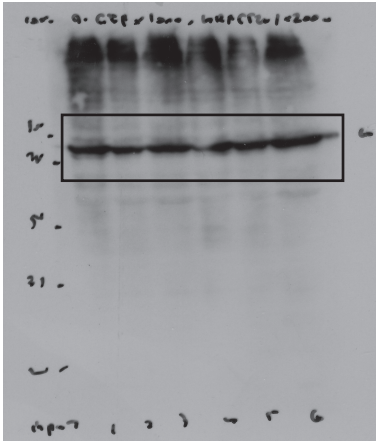

Source data for Fig. 5B

Supplement: Supplementary file 7 — Source data Fig. 5 [file 44318_2025_561_MOESM7_ESM.zip › Fig. 5/Source data for Fig. 5B/Source data for Fig. 5B.pdf]

Fig. 5C

$\alpha$ -ADP ribose

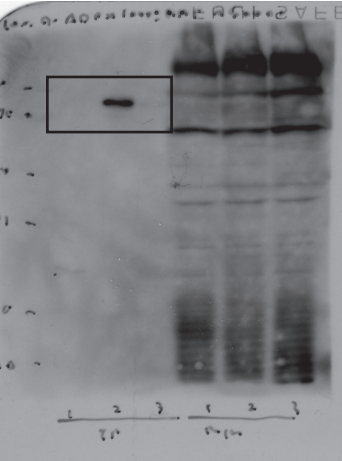

$\alpha$ -M2-FLAG

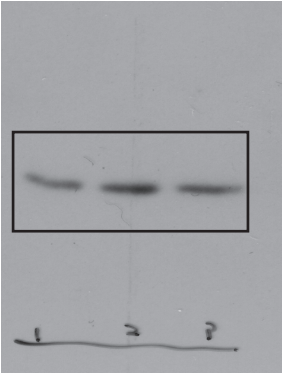

$\alpha$ -GFP (IP)

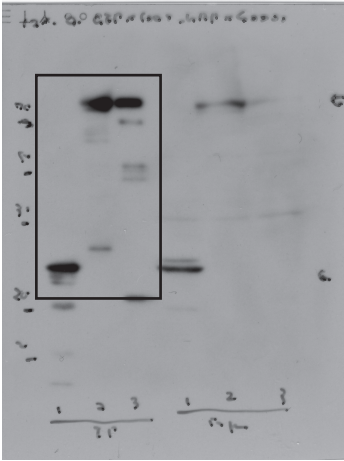

Source data for Fig. 5C

Supplement: Supplementary file 7 — Source data Fig. 5 [file 44318_2025_561_MOESM7_ESM.zip › Fig. 5/Source data for Fig. 5C/Source data for Fig. 5C.pdf]

Fig. 5D

$\alpha$ -cleaved casp8

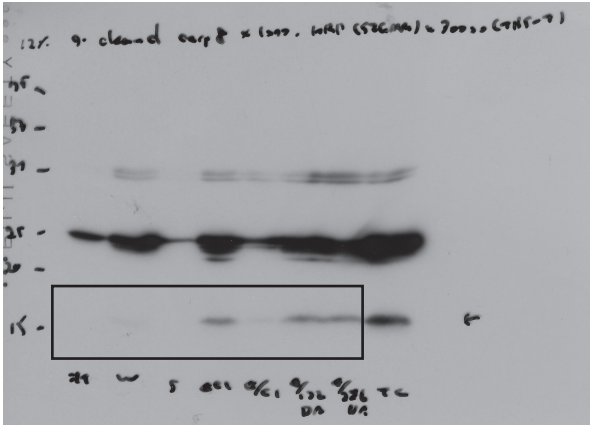

$\alpha$ -casp8 (mouse)

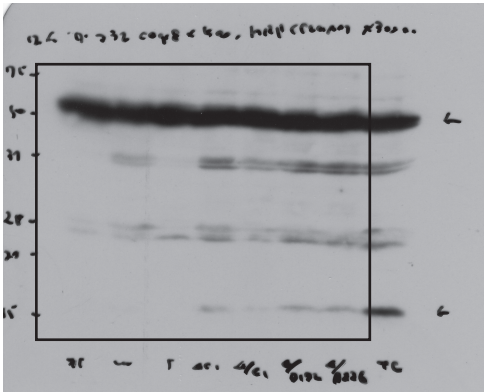

$\alpha$ -actin

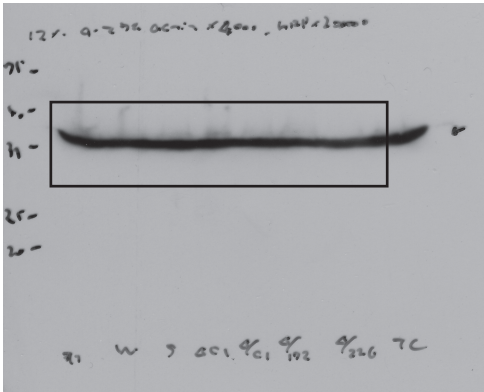

Source data for Fig. 5D

Supplement: Supplementary file 7 — Source data Fig. 5 [file 44318_2025_561_MOESM7_ESM.zip › Fig. 5/Source data for Fig. 5D/Source data for Fig. 5D.pdf]

Fig. 6B

$\alpha$ -p-MLKL

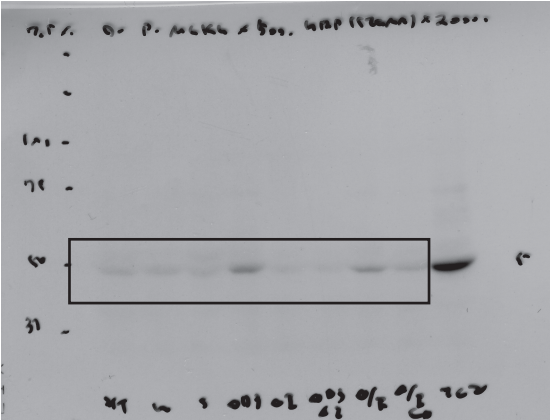

$\alpha$ -MLKL

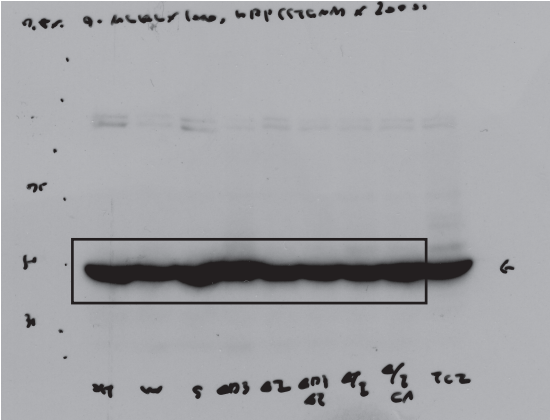

$\alpha$ -p-RIPK1

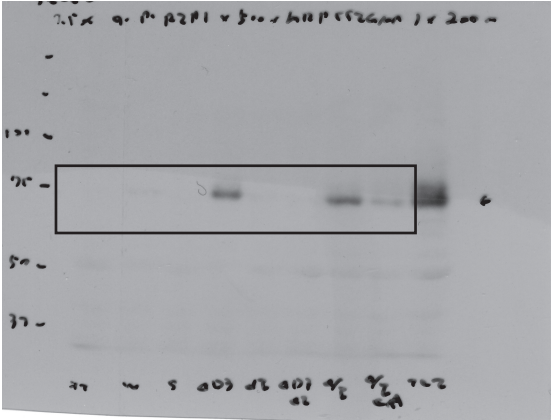

$\alpha$ -p-RIPK3

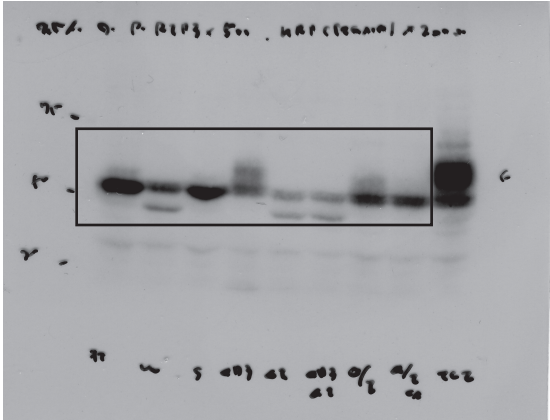

$\alpha$ -actin

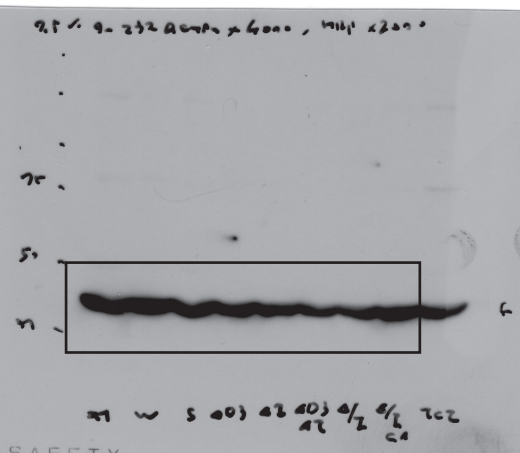

Source data for Fig. 6B

Supplement: Supplementary file 8 — Source data Fig. 6 [file 44318_2025_561_MOESM8_ESM.zip › Fig. 6/Source data for Fig. 6B/Source data for Fig. 6B.pdf]

Fig. 6D

$\alpha$ -p-MLKL

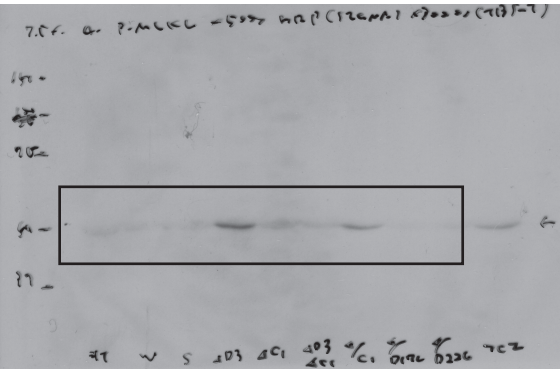

$\alpha$ -MLKL

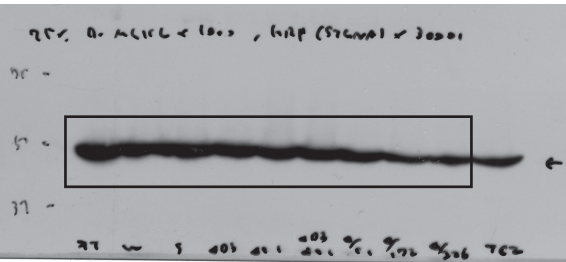

$\alpha$ -cleaved casp8

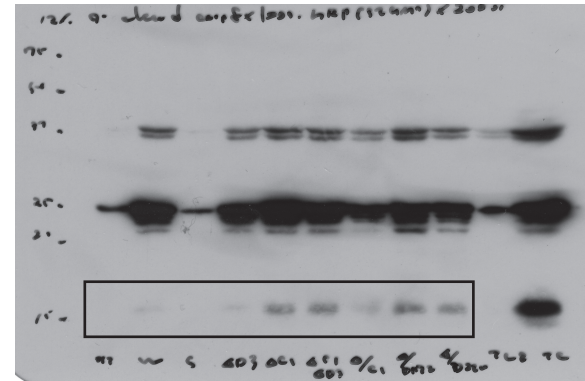

$\alpha$ -casp8 (Rb)

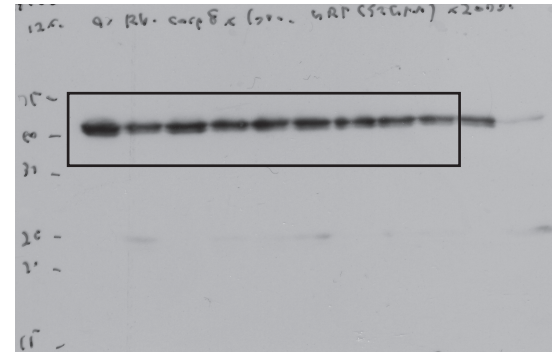

$\alpha$ -actin

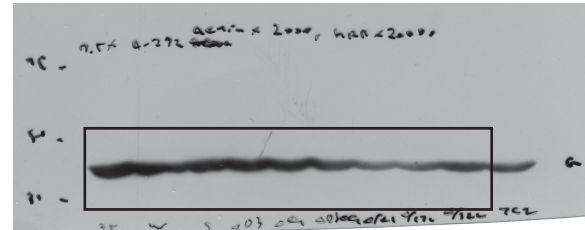

Source data for Fig. 6D

Supplement: Supplementary file 8 — Source data Fig. 6 [file 44318_2025_561_MOESM8_ESM.zip › Fig. 6/Source data for Fig. 6D/Source data for Fig. 6D.pdf]
