# Supplementary material for: Shigella type-III secretion system effectors counteract the induction of host inflammation and cell death
Source: EMBO J. 2025 Sep 10;44(21):6196–225. doi: 10.1038/s44318-025-00561-7 (PMC12583537; doi:10.1038/s44318-025-00561-7)
Supplement: Supplementary file 6 — Source data Fig. 4 [file 44318_2025_561_MOESM6_ESM.zip › Fig. 4/Source data for Fig. 4A/Source data for Fig. 4A.pdf]

Fig. 4A

α-cleaved casp8

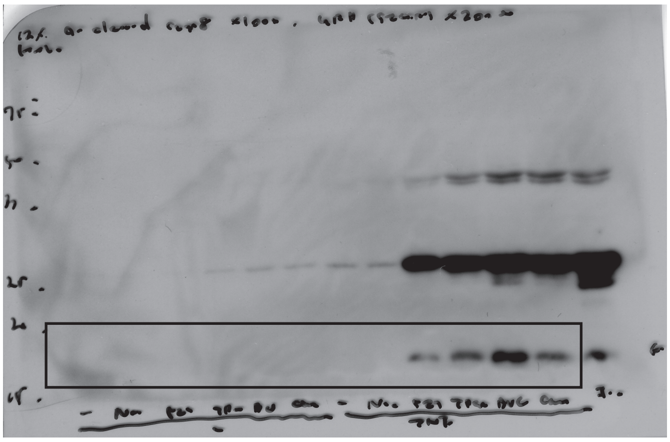

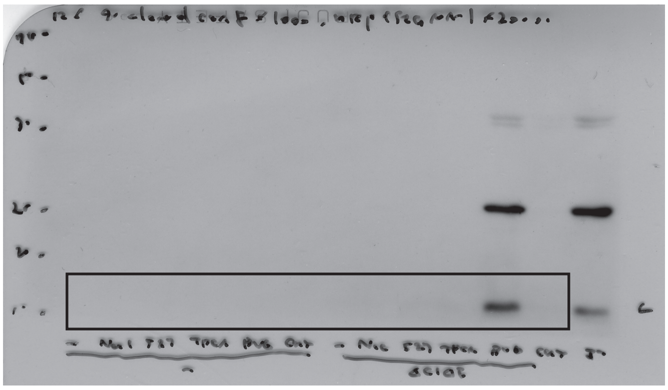

α-casp8 (mouse)

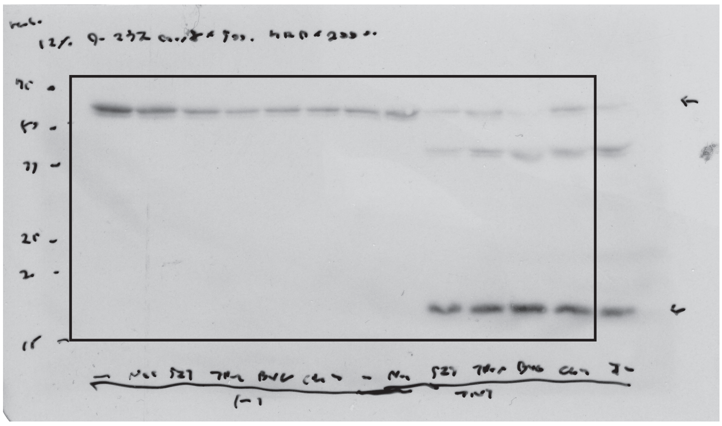

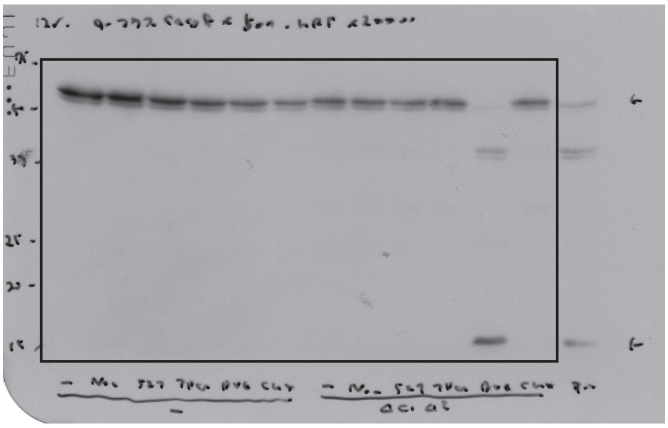

α-actin

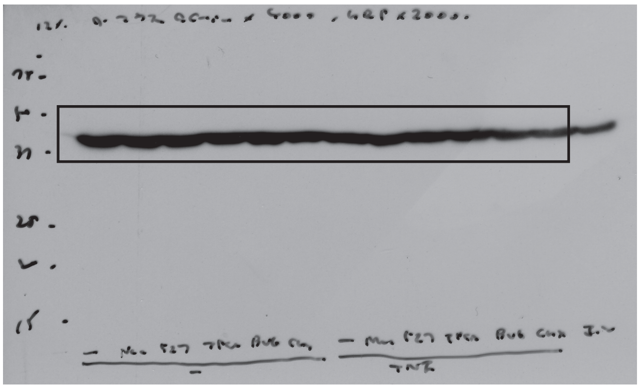

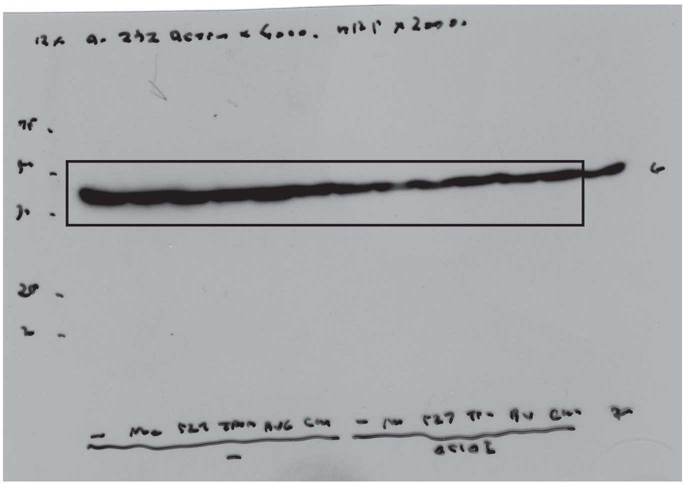

Source data for Fig. 4A
