# Supplementary material for: Shigella type-III secretion system effectors counteract the induction of host inflammation and cell death
Source: EMBO J. 2025 Sep 10;44(21):6196–225. doi: 10.1038/s44318-025-00561-7 (PMC12583537; doi:10.1038/s44318-025-00561-7)
Supplement: Supplementary file 9 — Expanded View Figures [file 44318_2025_561_MOESM9_ESM.pdf]

Expanded View Figures

Figure EV1. The *Shigella* effector OspI triggers caspase-8 activation.

(**A**, **B**) HeLa cells stably expressing OspI or OspI-C62A were stimulated with TNF-α (25 ng/mL), IL-1β (50 ng/mL), or PMA (50 μg/mL) and incubated for 8 h. Caspase-8 activity (**A**) or caspase-3 activity (**B**) was measured and is reported as relative light units (RLU) of stimulated samples normalized to the value in unstimulated samples. (**A**) Data are expressed as the mean ± SD from quadruplicate and representative of three independent experiments (P values: $P < 0.0001$ (left) and $P < 0.0001$ (right); two-way ANOVA). (**B**) Data are expressed as the mean ± SD from triplicate and representative of three independent experiments (P values: $P < 0.0001$ (left) and $P < 0.0001$ (right); two-way ANOVA). (**C**) HeLa cells stably expressing OspI or OspI-C62A were stimulated with TNF-α (25 ng/mL). At the indicated time points, cells were harvested and cell lysates were subjected to immunoblotting. Data are representative of three independent experiments. (**D**) HeLa cells stably expressing OspI or OspI-C62A were stimulated with TNF-α (10 ng/mL) or PMA (50 μg/mL) and incubated for 1 h. The expression level of the *IL8* gene was determined by real-time PCR. Relative expression change in gene expression was calculated using unstimulated cells as the control. Data are expressed as the mean ± SD from three independent experiments ($P = 0.8172$ for TNF-α and $P < 0.0001$ for PMA; two-way ANOVA). Data are considered significant when $P < 0.05$, with *$P < 0.05$ or n.s., not significant (**A**, **B**, **D**).

© The Author(s)

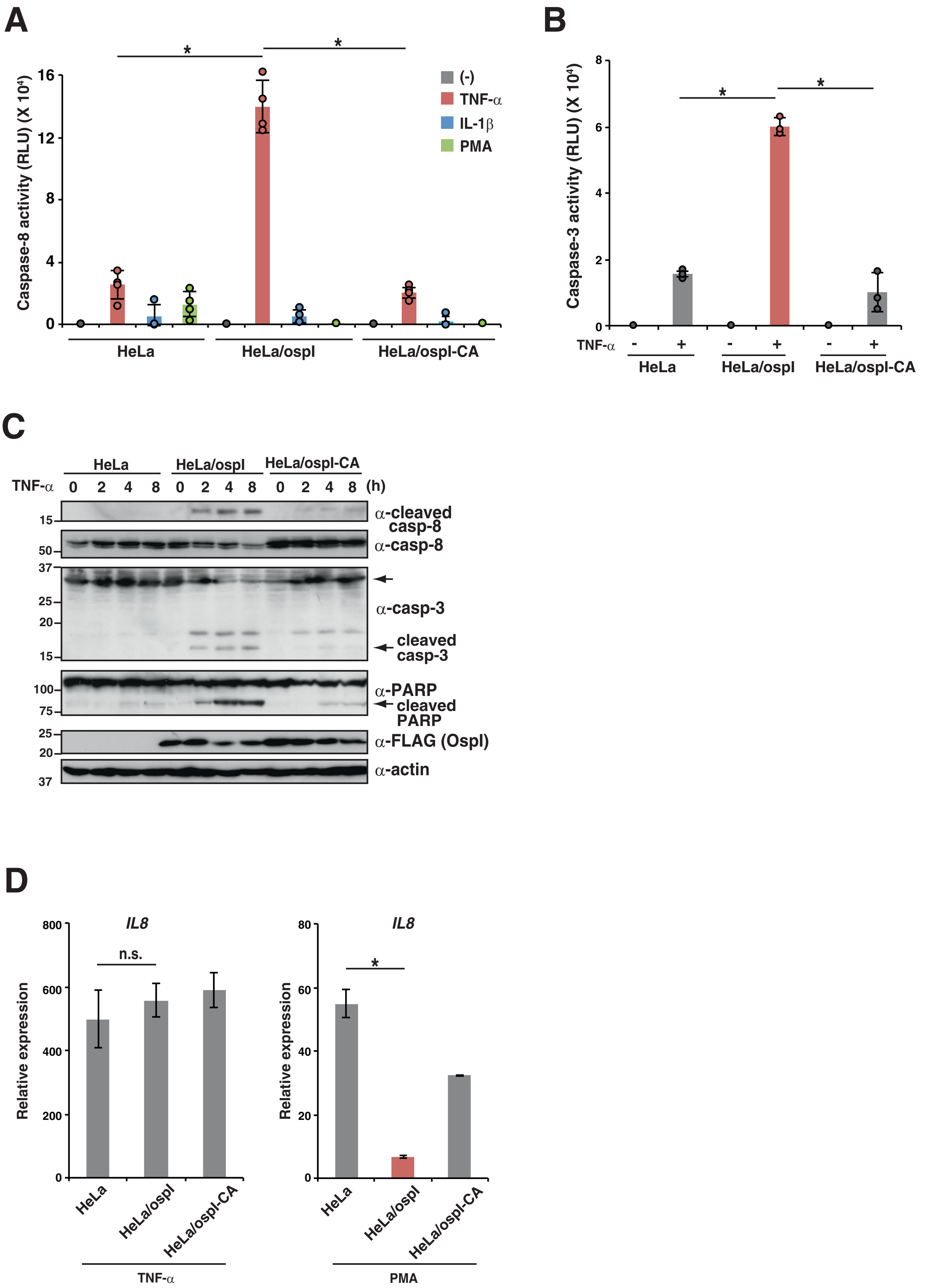
A
Caspase-8 activity (RLU) (X 10⁴)
0
4
8
12
16
*
*
(-)
TNF-α
IL-1β
PMA
HeLa
HeLa/ospI
HeLa/ospI-CA
B
Caspase-3 activity (RLU) (X 10⁴)
0
2
4
6
8
*
*
TNF-α
-
+
-
+
-
+
HeLa
HeLa/ospI
HeLa/ospI-CA
C
HeLa
HeLa/ospI
HeLa/ospI-CA
TNF-α 0 2 4 8 0 2 4 8 0 2 4 8 (h)
15
α-cleaved casp-8
50
α-casp-8
37
25
20
15
α-casp-3
cleaved casp-3
100
75
α-PARP
cleaved PARP
25
20
α-FLAG (OspI)
37
α-actin
D
IL8
Relative expression
0
200
400
600
800
n.s.
HeLa
HeLa/ospI
HeLa/ospI-CA
TNF-α
IL8
0
20
40
60
80
*
PMA

 © The Author(s)

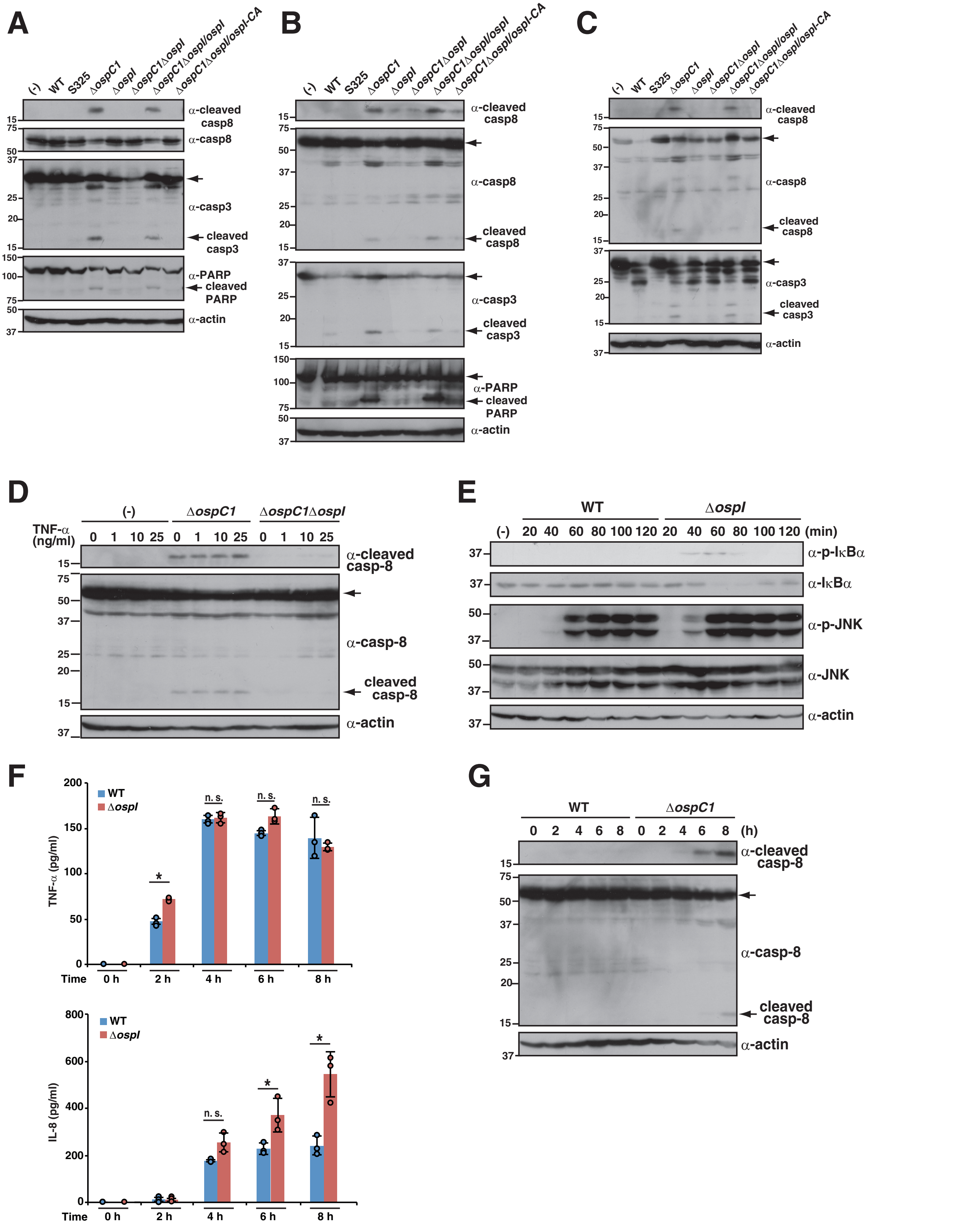
A
(-)
WT
S325
ΔospC1
ΔospI
ΔospC1ΔospI
ΔospC1ΔospI/ospI
ΔospC1ΔospI/ospI-CA
α-cleaved casp8
α-casp8
α-casp3
cleaved casp3
α-PARP
cleaved PARP
α-actin
B
α-cleaved casp8
α-casp8
cleaved casp8
α-casp3
cleaved casp3
α-PARP
cleaved PARP
α-actin
C
α-cleaved casp8
α-casp8
cleaved casp8
α-casp3
cleaved casp3
α-actin
D
TNF-α (ng/ml)
0 1 10 25 0 1 10 25 0 1 10 25
α-cleaved casp-8
α-casp-8
cleaved casp-8
α-actin
E
WT
ΔospI
(-) 20 40 60 80 100 120 20 40 60 80 100 120 (min)
α-p-IκBα
α-IκBα
α-p-JNK
α-JNK
α-actin
F
WT
ΔospI
TNF-α (pg/ml)
IL-8 (pg/ml)
n. s.
*
Time
0 h
2 h
4 h
6 h
8 h
G
WT
ΔospC1
0 2 4 6 8 0 2 4 6 8 (h)
α-cleaved casp-8
α-casp-8
cleaved casp-8
α-actin

Figure EV2. The *Shigella* effector OspI triggers caspase-8 activation.

(**A–C**) HeLa (**A**), HCT116 (**B**), or T84 (**C**) cells were infected with the indicated *Shigella* strains and incubated for 8 h (**A**), 6 h (**B**), or 12 h (**C**). Cell lysates were subjected to immunoblotting. (**D**) HT-29 cells were infected with the indicated *Shigella* strains in the presence or absence of TNF-α and incubated for 8 h. Cell lysates were then subjected to immunoblotting. (**E–G**) HT-29 cells were infected with the indicated *Shigella* strains. Cell lysates (**E**, **G**) or culture supernatants (**F**) obtained at the indicated time points were subjected to immunoblotting (**E**, **G**) or ELISA (**F**). (**F**) Data are expressed as the mean ± SD from triplicate and representative of three independent experiments (TNF-α : $P = 0.0384$ for 2 h, $P > 0.9999$ for 4 h, $P = 0.2229$ for 6 h, and $P = 0.8763$ for 8 h. IL-8: $P = 0.4407$ for 4 h, $P = 0.0129$ for 6 h, and $P < 0.0001$ for 8 h; two-way ANOVA). Data are representative of three independent experiments (**A–E**, **G**). Molecular weights in immunoblots are in kDa. Data are considered significant when $P < 0.05$, with $^*P < 0.05$ or n.s., not significant (**F**).

 © The Author(s)

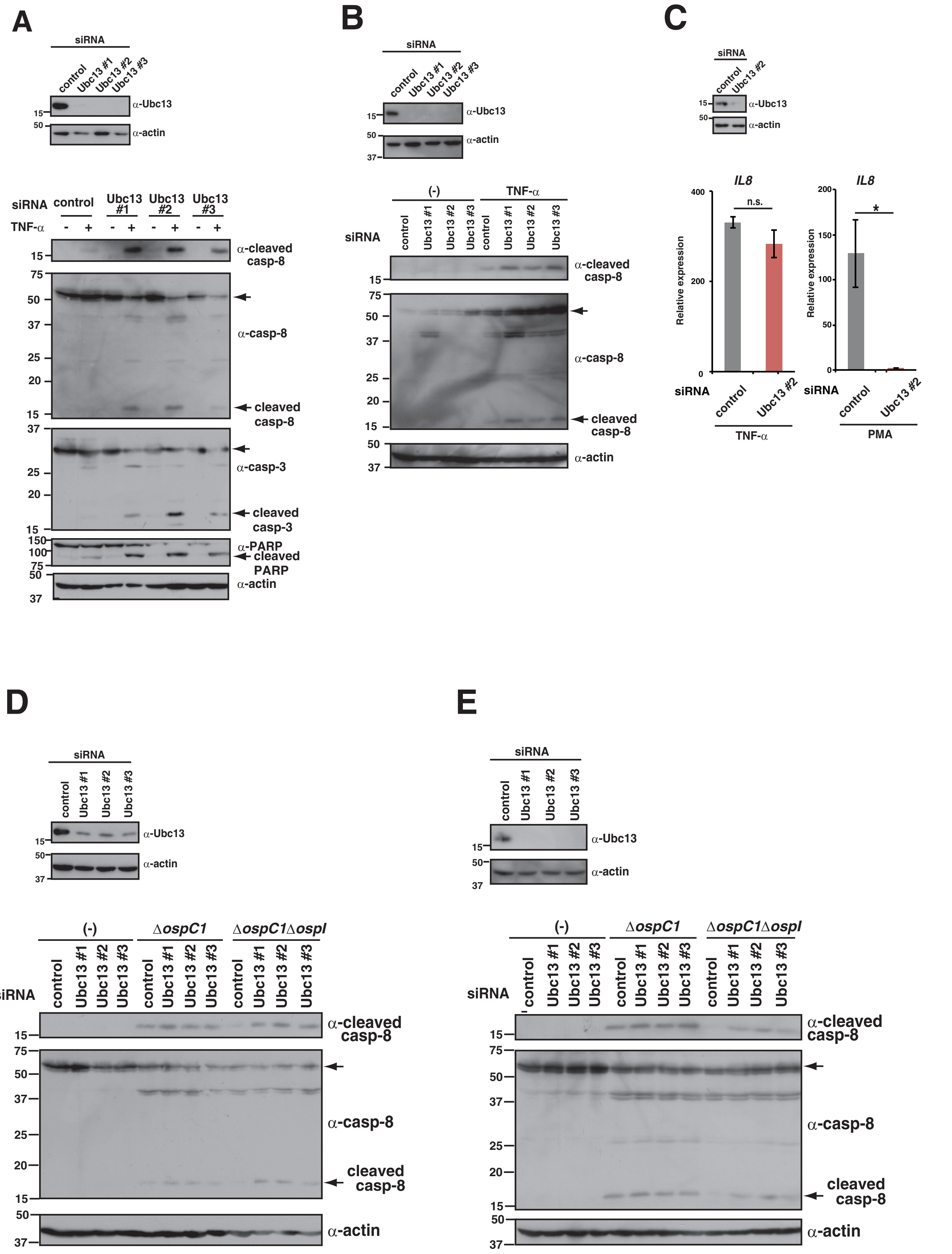
A
B
C
D
E
siRNA
control
Ubc13 #1
Ubc13 #2
Ubc13 #3
α-Ubc13
α-actin
TNF-α
α-cleaved casp-8
α-casp-8
cleaved casp-8
α-casp-3
cleaved casp-3
α-PARP
cleaved PARP
(-)
IL8
n.s.
*
Relative expression
PMA
ΔospC1
ΔospC1ΔospI

Figure EV3. Ubc13 inactivation triggers caspase-8 activation under TNF-α stimulation.

(**A**, **B**) HeLa (**A**) or HCT116 (**B**) cells treated with control or Ubc13 siRNAs were stimulated with TNF-α (25 ng/mL) and incubated for 8 h. Cell lysates were subsequently subjected to immunoblotting. The knockdown efficiency of the indicated siRNAs was assessed by immunoblotting (inset). (**C**) HeLa cells treated with control or Ubc13 siRNAs were stimulated with TNF-α (10 ng/mL) or PMA (50 µg/mL) and incubated for 1 h. The expression level of the *IL8* gene was determined by real-time PCR. Relative expression change in gene expression was calculated using unstimulated cells as the control. Data are expressed as the mean ± SD from three independent experiments ($P = 0.2176$ for TNF-α and $P = 0.0278$ for PMA; two-tailed Student's *t* test). The knockdown efficiency of the indicated siRNAs was assessed by immunoblotting (inset). (**D**, **E**) HeLa (**D**) or HCT116 (**E**) cells treated with control or Ubc13 siRNAs were infected with the indicated *Shigella* strains and incubated for 8 h. Cell lysates were subjected to immunoblotting. The knockdown efficiency of the indicated siRNAs was assessed by immunoblotting (inset). Data are representative of three independent experiments (**A**, **B**, **D**, **E**). Molecular weights in immunoblots are in kDa. Data are considered significant when $P < 0.05$, with $^*P < 0.05$ or n.s., not significant (**C**).

© The Author(s)

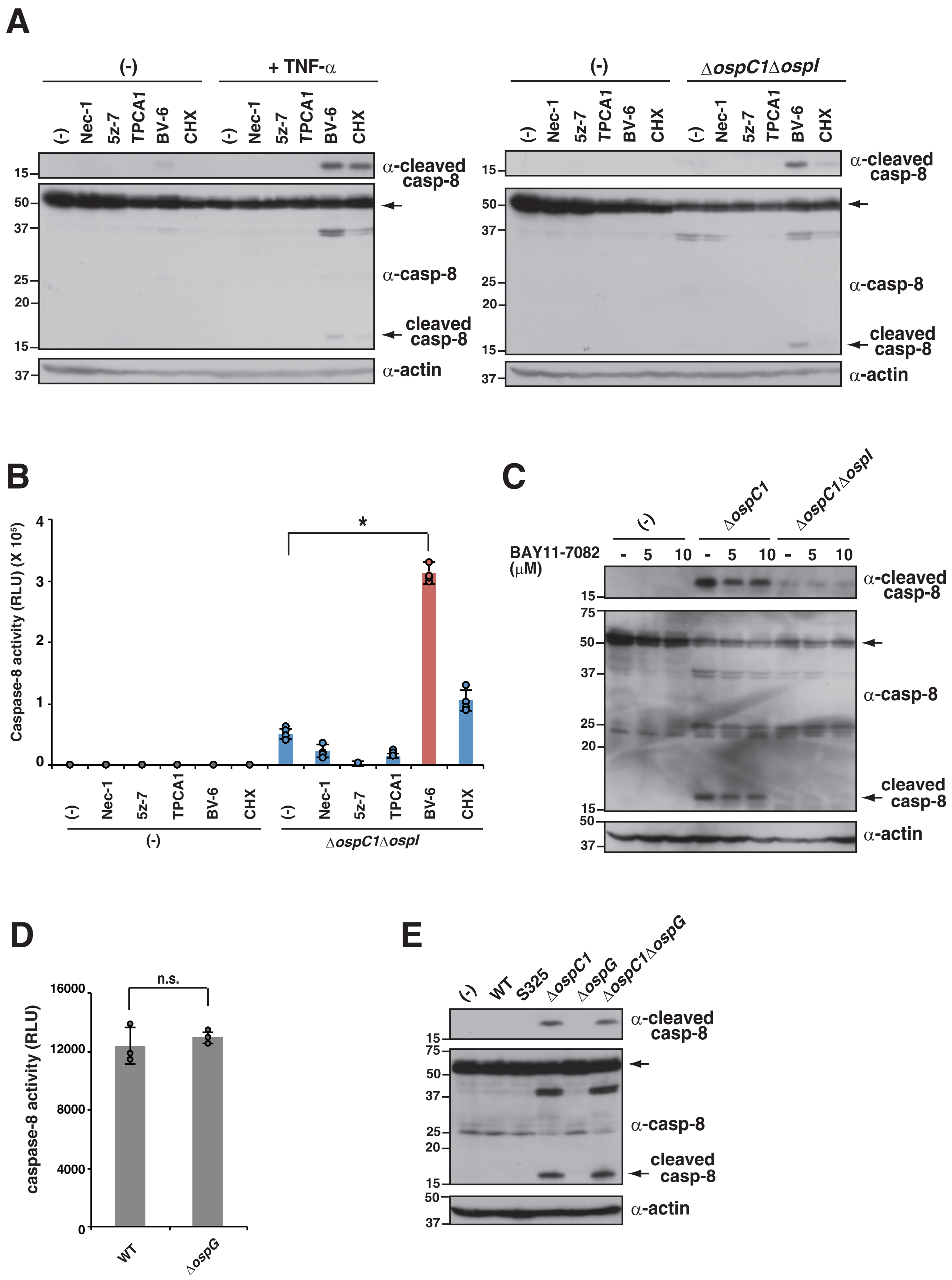
A
(-)
+ TNF-α
(-) Nec-1 5z-7 TPCA1 BV-6 CHX
α-cleaved casp-8
α-casp-8
cleaved casp-8
α-actin
ΔospC1ΔospI
B
Caspase-8 activity (RLU) (X 10⁵)
*
C
BAY11-7082 (μM)
- 5 10
ΔospC1
D
caspase-8 activity (RLU)
n.s.
WT
ΔospG
E
S325
ΔospC1ΔospG

◀ **Figure EV4. *Shigella* effector OspI-mediated IAP inactivation triggers caspase-8 activation.**

(**A**, **B**) HT-29 cells were stimulated with TNF-α (25 ng/mL) or infected with *Shigella ΔospC1ΔospI* in the presence or absence of Nec-1 (50 μM), 5z-7 (0.5 μM), TPCA1 (5 μM), BV-6 (1 μM), or CHX (25 μg/mL) and incubated for 8 h. Cell lysates were subjected to immunoblotting (**A**) or measurement of caspase-8 activity (**B**). Caspase-8 activity is reported in terms of relative light units (RLUs) of infected samples normalized to the values in uninfected samples. Data are expressed as the mean ± SD from quadruplicate and representative of three independent experiments (P value: $P < 0.0001$; two-way ANOVA). (**C**) HT-29 cells were infected with the indicated *Shigella* strains in the presence or absence of a BAY11-7082 inhibitor (5 or 10 μM) and incubated for 8 h. Cell lysates were subjected to immunoblotting. (**D**) HT-29 cells were infected with the indicated *Shigella* strains and incubated for 8 h. Cells were then harvested and caspase-8 activity was measured. Caspase-8 activity is reported as RLUs of infected samples normalized to the values in uninfected samples. Data are expressed as the mean ± SD from triplicate and representative of three independent experiments ($P = 0.52$; two-tailed Student's t test). (**E**) HT-29 cells were infected with indicated *Shigella* strains and incubated for 8 h. Cell lysates were subjected to immunoblotting. Data are representative of three independent experiments (**A**, **C**, **E**). Molecular weights in immunoblots are in kDa. Data are considered significant when $P < 0.05$, with $*P < 0.05$ or n.s., not significant (**B**, **D**).

 © The Author(s)

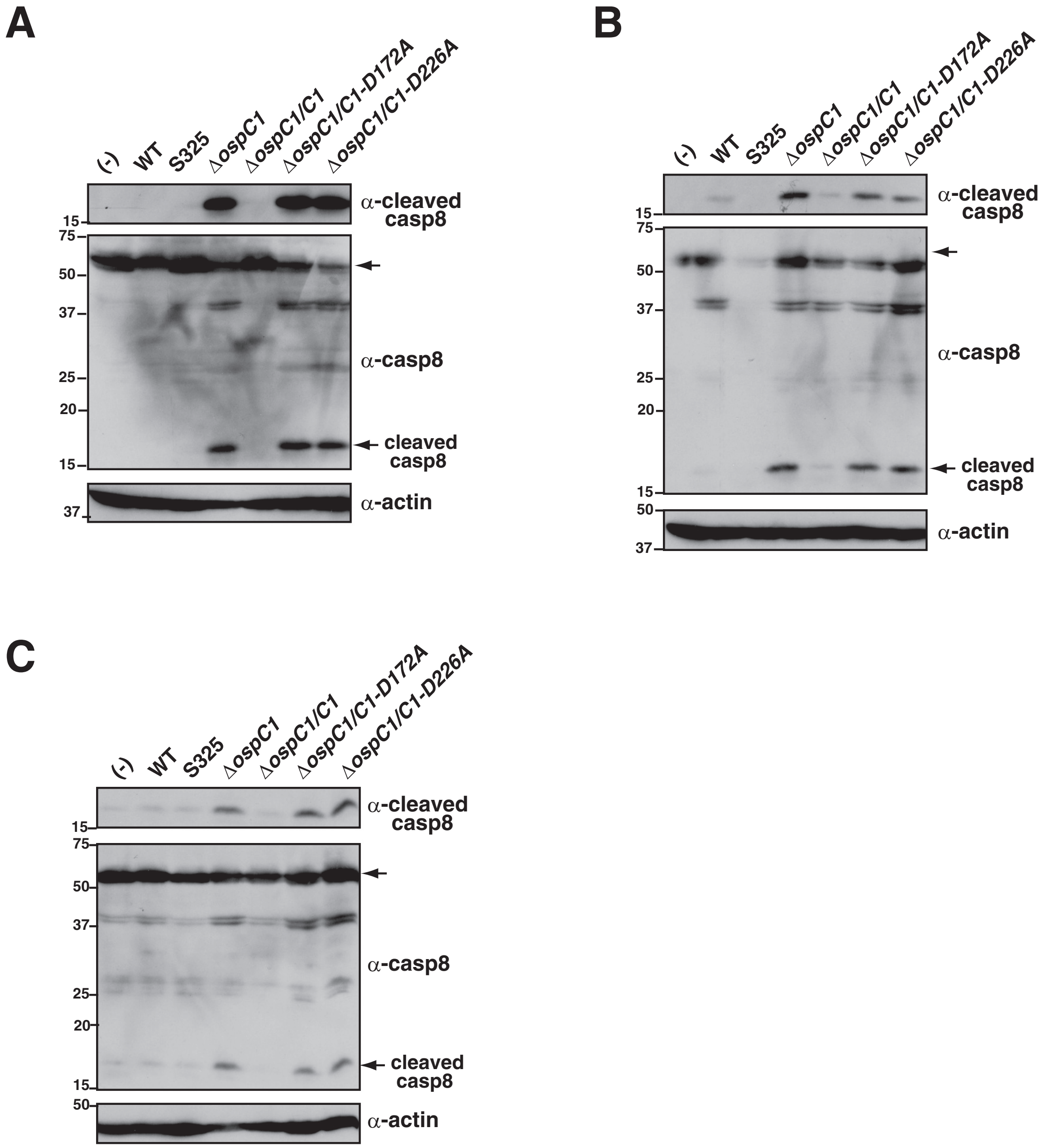

Figure EV5. The ADP-riboxanation activity of OspC1 is required for caspase-8 inhibition.

(**A–C**) HeLa (**A**), HCT116 (**B**) or T84 (**C**) cells were infected with *Shigella* WT, S325, *ΔospC1*, *ΔospC1/ospC1* (*ΔospC1* complemented with *ospC1*), *ΔospC1/ospC1-D172A* (*ΔospC1* complemented with *ospC1-D172A*), or *ΔospC1/ospC1-D226A* (*ΔospC1* complemented with *ospC1-D226A*) strains and incubated for 6 h (**A**, **B**) or 12 h (**C**). Cell lysates were subjected to immunoblotting. All data are representative of three independent experiments. Molecular weights in immunoblots are in kDa.

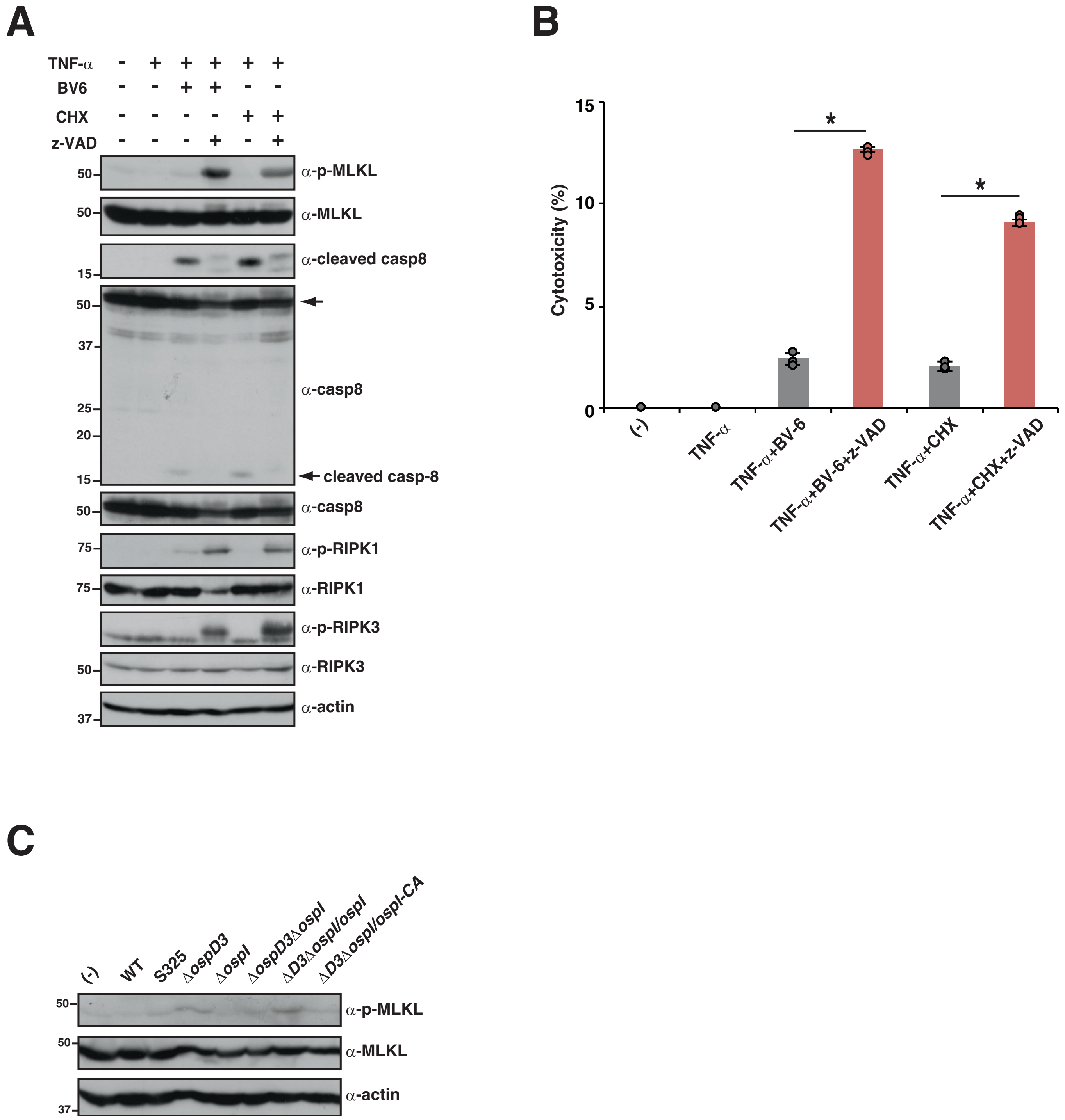

Figure EV6. Caspase-8 is the molecular switch for apoptosis and necroptosis.

(**A**, **B**) HT-29 cells were treated with TNF-α (25 ng/mL) plus the indicated inhibitors (BV-6, 1 μM; CHX, 25 μg/mL; Z-VAD-fmk, 10 μM) and incubated for 8 h. Cell lysates and aliquots of cellular supernatants were subjected to immunoblotting (**A**) or cytotoxicity assays (**B**), respectively. (**B**) Data are expressed as the mean ± SD from triplicate and representative of three independent experiments (*P* values: $P < 0.0001$ (left) and $P < 0.0001$ (right); two-way ANOVA). (**C**) HT-55 cells were infected with the indicated *Shigella* strains and incubated for 12 h before cell lysates were subjected to immunoblotting. Data are representative of three independent experiments (**A**, **C**). Molecular weights in immunoblots are in kDa. Data are considered significant when $P < 0.05$, with $^{*}P < 0.05$ (**B**).

 © The Author(s)
